# Metformin effects on respiratory and metabolic outcomes in asthma and metabolic syndrome: a double-blind, randomized, placebo-controlled clinical trial

**DOI:** 10.1097/MS9.0000000000003552

**Published:** 2025-07-18

**Authors:** Hossein Mehravaran, Adeleh Bahar, Fatemeh Hajimohammadi, Zahra Kashi, Masoud Aliyali, Fatemeh Varshoei, Reza Alizadeh-Navaei, Jamshid Yazdani Charati, Alireza Kashefizadeh, Mobina Gheibi, Erfan Ghadirzadeh

**Affiliations:** a Division of Pulmonary and Critical Care, Department of Internal Medicine, School of Medicine, Mazandaran University of Medical Sciences, Sari, Iran; b Diabetes Research Center, Mazandaran University of Medical Sciences, Sari, Iran; c Student Research Committee, Faculty of Medicine, Mazandaran University of Medical Sciences, Sari, Iran; d Department of Pediatrics, School of Medicine, Mazandaran University of Medical Sciences, Sari, Iran; e Non-communicable Diseases Institute, Mazandaran University of Medical Sciences, Sari, Iran; f Department of Biostatistics and Epidemiology, School of Health, Health Sciences Research Center, Mazandaran University of Medical Sciences, Sari, Iran; g Department of Pulmonology, Shahid Labbafinejad Hospital, Shahid Beheshti University of Medical Sciences, Tehran, Iran

**Keywords:** asthma, clinical trial, inflammation, metabolic syndrome, metformin

## Abstract

**Introduction:**

Metabolic syndrome (MetS) could be associated with worsened asthma control and more severe and frequent asthma attacks. Metformin has been suggested as a potential treatment for MetS, which could decrease airway reactivity and lead to improved asthma control and reduced emergency department (ED) visits. However, the existing data have predominantly focused on diabetic patients only. Thus, in the present study, we aimed to investigate the effects of metformin in patients with concurrent asthma and MetS.

**Methods:**

This trial was conducted on two groups of patients (55 in each group) with concurrent asthma and MetS, each receiving either metformin hydrochloride or an identical placebo. Patients were followed up after 3 months and assessed regarding clinical outcomes, asthma control tests (ACTs), pulmonary function test parameters, C-reactive protein (CRP) levels, frequency of asthma attacks, ED visits, and hospitalization rate.

**Results:**

The metformin group showed statistically significant improvements in indices regarding ACT score, O_2_ saturation, anthropometric indices, blood sugar control, CRP, and lipid profile compared to the placebo group (all *P* < 0.05). Additionally, the metformin group showed significantly higher forced expiratory volume in 1 s (FEV_1_) (*P* = 0.014, and effect size = 5.6%) and forced vital capacity (FVC) (*P* = 0.001, and effect size = 9.2%) in the multivariate analysis after controlling for the confounding effects of baseline parameters and sex. Although the metformin group demonstrated a trend toward a reduction in severe asthma attacks, ED visits, or hospitalization rates, the effects were not statistically significant (*P* > 0.05).

**Conclusion:**

Our findings demonstrate that metformin administration in patients with concurrent asthma and MetS could lead to substantial enhancements in ACT score, FEV_1_, and FVC.

**HIGHLIGHTS**

- Metabolic syndrome is associated with worsened asthma control.
- Metabolic syndrome is associated with severe and frequent asthma attacks.
- Metformin administration can enhance the pulmonary function in asthma/metabolic syndrome patients.

## Introduction

Asthma is a serious worldwide health issue influencing all age groups^[1]^. It is estimated that almost 235 million individuals suffer from asthma worldwide, with the highest prevalence in high-income countries with increasing incidence^[2]^. Metabolic syndrome (MetS) consists of a group of symptoms, including dyslipidemia, abdominal obesity, hypertension, chronic inflammatory state, insulin resistance, and elevated blood sugar (BS) levels, which serve as risk factors for type 2 diabetes mellitus (T2DM) and coronary artery disease^[3]^. It has been estimated that over a billion individuals are affected by MetS worldwide^[4]^. Recent studies have shown a 32.5% prevalence of MetS among the US population in 2011, up to 36.9% in 2016, showing a significant increasing trend in prevalence, especially in Asian ethnicity living in the USA^[5]^.

Many studies have shown that MetS may also lead to an increased risk of comorbidities such as asthma^[6]^. In addition, it has been shown that MetS could be associated with worsened asthma control and more severe and frequent asthma attacks^[7]^. Although possible mechanisms have been proposed to explain the association between asthma and MetS, including oxidative stress, inflammatory cytokines released from adipose tissue, and the mechanical effects of obesity on pulmonary function, the pathogenesis remains poorly understood^[8-10]^.

With the increasing prevalence of both conditions, it is essential to identify medications that benefit both. Metformin, a dimethylbiguanidine, has been suggested as a potential treatment for MetS, which could reduce central obesity, promote BS control, and reduce inflammatory responses^[11–13]^. Additionally, metformin may decrease airway reactivity by activating the AMP-activated protein kinase (AMPK) and the miR-152-3p/DNMT1 axis, leading to improved asthma control and a reduction in emergency department (ED) visits in asthmatic patients^[14,15]^. However, the existing data are either primarily conducted on rat models or are observational studies that have predominantly focused on the effects of metformin on asthma control and its outcomes in diabetic patients only^[16]^. Therefore, it remains unclear whether the effects of metformin on asthma are restricted to patients with DM or whether it may also benefit subjects with MetS and can be started in this population, especially if it is in association with asthma^[17]^. In the present study, we aimed to investigate the effects of metformin on clinical outcomes, asthma control tests (ACTs), pulmonary function tests (PFTs), BS control, and C-reactive protein (CRP) levels in patients with concurrent asthma and MetS.

## Materials and methods

### Study design, site, population, and duration

This double-blind, randomized, placebo-controlled trial was conducted on adult patients with concurrent asthma and MetS admitted to the ED of a tertiary care hospital in north of Iran, between November 2022 and June 2023, due to a severe asthma attack. Our study was conducted in northern Iran, a region characterized by a diverse population, cultures, and environments. This geographical diversity includes different ethnicities and genetic backgrounds (including Persians and Afghans), as well as various geographical environments, including both mountainous areas and seashores, contributing to a varied environmental context.

### Inclusion and exclusion criteria

A respiratory consultant confirmed the diagnosis of asthma based on the patient’s typical clinical symptoms and signs and a review of previous spirometric records of at least 12% and 200 mL bronchodilator reversibility results in accordance with GINA guidelines^[18]^. An endocrine physician consultant confirmed the diagnosis of MetS according to the National Cholesterol Education Program Adult Treatment Panel III (NCEP ATP III) definition. Based on these criteria, MetS is defined by the presence of at least three of the following criteria: (1) waist circumference ≥ 88 cm in female patients and ≥102 in male patients, (2) serum triglyceride (TG) ≥ 150 mg/dL (or on TG-lowering medical therapy), (3) serum high-density lipoprotein (HDL) < 40 mg/dL in male patients and <50 mg/dL in female patients, (4) blood pressure (BP) ≥ 130/85 mmHg (or on BP-lowering medical therapy), and (5) fasting BS (FBS) ≥ 100 mg/dL (or on BS-lowering medical therapy)^[19]^.

The minimum required sample size in each group was calculated as 55 considering *a* = 0.05, *B* = 0.2, and *r* = 0.37 based on the Gutiérrez-Carrasquilla *et al*^[20]^ study. Patients with concurrent asthma and MetS diagnoses were included, and patients with chronic obstructive pulmonary disease (COPD), hypercapnic respiratory failure, bronchiectasis, cancer, T2DM (or on BS-lowering medication), malnutrition, renal failure (with an estimated glomerular filtration rate of less than 30 mL/min), class IV heart failure, thalassemia major and intermedia, sickle cell anemia, severe liver failure or sepsis, patients already on oral corticosteroid medications before admission, patients with serum procalcitonin level more than 0.25 ng/mL, connective tissue diseases, and patients not willing to participate were excluded.

### Primary and secondary outcome measurements, randomization, and intervention

At admission, demographic data, vital signs, and relevant clinical features were recorded, and blood samples were taken for laboratory analysis, including BS, hemoglobin A1c (HbA1c), creatinine (Cr), TG, HDL, low-density lipoprotein (LDL), total cholesterol (Chol), CRP as an inflammatory marker, and serum procalcitonin level. PFT was performed using GANSHORN Spiroscout (GANSHORN Schiller Group, Germany).

Using block randomization (in blocks of 4, as determined by the sealed envelope website), patients were randomly allocated into two groups: placebo and intervention. The intervention consisted of 500 mg metformin hydrochloride (Koushan Pharmed) on the first 2 days and 1000 mg thereafter, until the end of the third month. The placebo group received identical metformin tablets without any active ingredients until the end of the study period. All patients received standard treatment for severe asthma attacks, according to the GINA guidelines^[18]^. Patients, their care providers, and outcome assessors were all blinded in this trial. All patients were on a regular diet and engaged in physical activity, and no significant dietary or exercise interventions were considered for them. Patients were followed up and re-evaluated in the third month. If there was a history of an asthma attack episode, ED visit, or hospitalization during the 3-month follow-up period, researchers contacted the hospital or clinic to obtain and confirm the information. All measurements and an ACT score were assessed and recorded in the third month. The ACT is a self-administered questionnaire comprising five items with a 4-week recall period. Scores range from 5 to 25, with higher values indicating enhanced control (≥20 denotes well-controlled asthma)^[21]^.

### Statistics

Describing quantitative and qualitative variables was done using mean ± standard deviation and frequency tables, respectively. Additionally, using a paired samples *t*-test, variables were compared between the admission time and the third month within each group to identify trends specific to each group. To perform quantitative comparisons between the groups, an independent samples *t*-test was conducted. For qualitative comparisons between the two groups, the chi-squared test was utilized. At both admission and the third month, comparisons were conducted between the two groups. Analysis of covariance (ANCOVA) with Bonferroni correction was applied after controlling for each variable’s baseline parameter and sex as covariates. In this study, data analysis was carried out using SPSS version 22 software, and a significant level was considered: *P* < 0.05. Additionally, since both forced expiratory volume in 1 s (FEV_1_) and forced vital capacity (FVC) are variables that assess lung function and are correlated with each other, we conducted a multivariate analysis to compare these two pulmonary function indices between groups simultaneously.

### Ethics

This study was conducted without commercial input or involvement in the design, implementation, analysis, or reporting. All procedures performed in this study were in accordance with the ethical standards of the Institutional Research Ethics Committee and with the 1964 Helsinki Declaration and its later amendments or comparable ethical standards. Written informed consent was obtained from all participants prior to their participation in the study.

## Results

**Figure 1.**
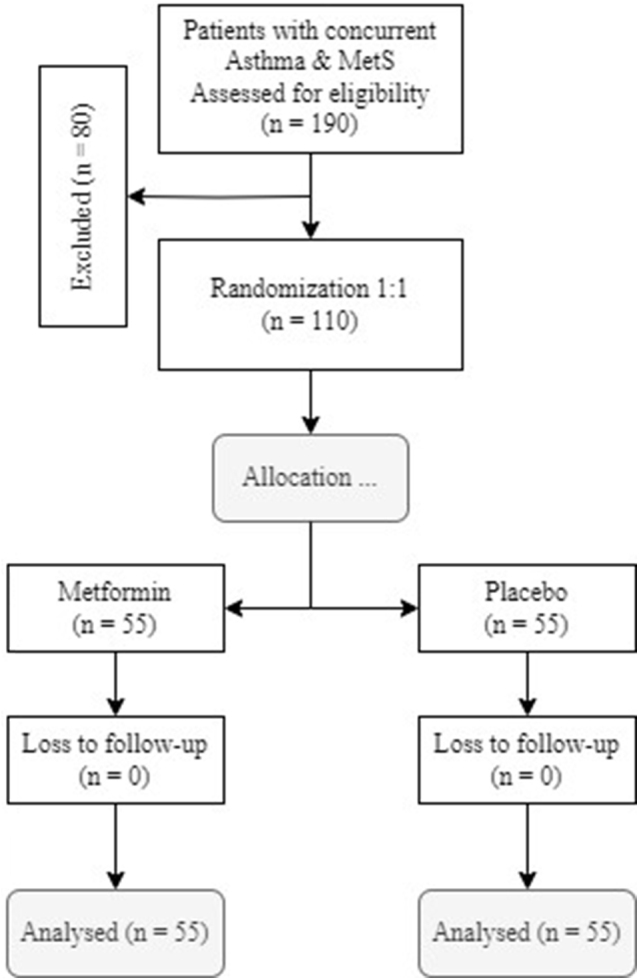
CONSORT clinical trial flowchart.

In this trial, 190 patients with concurrent asthma and MetS were assessed for eligibility, of which 80 met the exclusion criteria. Ultimately, 110 patients were included in the study. They were randomly assigned to two groups of 55, receiving either metformin or a placebo, and were followed up for 3 months. Figure 1 shows a flow chart of the present study. The two groups did not differ significantly in terms of baseline characteristics or baseline asthma medications (Table 1). Additionally, the two groups did not differ regarding any of the study variables at admission (all *P*-values > 0.05) (Table 2).

**Table 1:** Baseline characteristics of patients with asthma and Met syndrome

Variable	Metformin	Placebo	*P*-value*
Age	56.38 ± 11.013	55.02 ± 10.494	0.508
Gender			1.000
Male	26 (47.3%)	26 (47.3%)	
Female	29 (52.7%)	29 (52.7%)	
Past medical history			
HF	5 (9.1%)	4 (7.3%)	1.000
MI	11 (20%)	8 (14.5%)	0.615
CVA	4 (7.3%)	4 (7.3%)	1.000
Smoker	17 (30.9%)	15 (27.3%)	0.834

CVA, cerebrovascular accident; HF, heart failure; MI, myocardial infarction.

^*^ A *P*-value of less than 0.05 was considered statistically significant.

**Table 2:** Clinical, Respiratory and Metabolic parameters of Patients with Asthma and MetS

Variables	At admission	Month 3	Mean difference (95% CI)	*P*-value**	Effect size (%)**
BMI
Met	29.10 ± 1.68	28.38 ± 1.67	−0.50 (−0.65, −0.34)	<0.001	19.5
Pl	28.97 ± 1.75	28.75 ± 1.71			
*P*-value*	0.682	0.254			
WHR
Met	0.92 ± 0.02	0.88 ± 0.02	−0.02 (−0.03, −0.02)	<0.001	47
Pl	0.92 ± 0.02	0.91 ± 0.02			
*P*-value*	0.624	<0.001			
SBP
Met	147.29 ± 7.94	135.00 ± 4.07	−2.60 (−3.78, −1.42)	<0.001	11.2
Pl	147.15 ± 7.10	137.55 ± 4.79			
*P*-value*	0.920	0.003			
DBP
Met	96.38 ± 6.37	85.76 ± 3.08	−2.59 (−3.62, −1.57)	0.023	4.8
Pl	96.62 ± 5.72	88.44 ± 3.46			
*P*-value*	0.838	<0.001			
HR
Met	121.16 ± 7.24	75.27 ± 4.88	−1.60 (−3.29, .09)	0.348	0.8
Pl	121.42 ± 6.74	76.91 ± 4.63			
*P*-value*	0.849	0.074			
RR
Met	32.49 ± 1.68	12.82 ± 0.84	−0.56 (−0.92, −0.19)	0.083	2.8
Pl	32.64 ± 1.69	13.40 ± 1.09			
*P*-value*	0.653	0.002			
SpO_2_
Met	89.56 ± 0.93	97.33 ± 0.64	0.35 (0.12, 0.57)	0.009	6.3
Pl	89.71 ± 1.04	97.04 ± 0.76			
*P*-value*	0.445	0.033			
BS
Met	109.31 ± 3.17	94.27 ± 2.99	−9.05 (−9.93, −8.16)	<0.001	62.6
Pl	109.00 ± 2.43	103.13 ± 2.86			
*P*-value*	0.568	<0.001			
HbA1c
Met	6.20 ± 0.13	5.78 ± 0.12	−0.29 (−0.32, −0.25)	<0.001	52.7
Pl	6.20 ± 0.12	6.07 ± 0.14			
*P*-value*	0.854	<0.001			
CRP
Met	41.11 ± 9.94	5.22 ± 2.46	−5.83 (−7.07, −4.60)	<0.001	30.4
Pl	41.91 ± 10.06	11.22 ± 4.79			
*P*-value*	0.676	<0.001			
TG
Met	224.44 ± 22.27	204.09 ± 19.83	−11.22 (−13.28, −9.17)	<0.001	34.9
Pl	223.35 ± 20.50	214.35 ± 19.88			
*P*-value*	0.790	0.008			
HDL
Met	38.80 ± 3.74	43.95 ± 3.53	2.77 (2.20, 3.33)	<0.001	30.4
Pl	37.89 ± 3.41	40.35 ± 3.54			
*P*-value*	0.186	<0.001			
LDL
Met	127.59 ± 13.53	112.15 ± 13.29	−8.04 (−9.52, −6.57)	<0.001	33.2
Pl	126.85 ± 12.80	119.51 ± 12.10			
*P*-value*	0.771	0.003			
Chol
Met	211.31 ± 15.30	196.89 ± 14.35	−7.14 (−8.37, −5.92)	<0.001	38.4
Pl	209.96 ± 15.18	202.82 ± 1405			
*P*-value*	0.644	0.031			
FEV_1_
Met	51.04 ± 5.70	74.47 ± 3.46	1.74 (0.34, 3.14)	0.171	1.8
Pl	51.75 ± 5.73	72.89 ± 4.34			
*P*-value*	0.517	0.037			
FVC
Met	64.87 ± 5.06	79.64 ± 2.49	1.51 (0.54, 2.48)	0.041	3.9
Pl	64.89 ± 5.37	78.13 ± 2.95			
*P*-value*	0.985	0.005			
FEV_1_/FVC
Met	54.64 ± 5.56	72.47 ± 2.98	1.27 (0.12, 2.43)	0.112	2.4
Pl	55.07 ± 5.76	71.29 ± 3.58			
*P*-value*	0.687	0.063			

BMI, body mass index; BS, blood sugar; Chol, total cholesterol; CRP, C-reactive protein; DBP, diastolic blood pressure; FEV_1_, forced expiratory volume in 1 s; FVC, forced vital capacity; HbA1c, hemoglobin A1c; HDL, high-density lipoprotein; HR, heart rate; LDL, low-density lipoprotein; RR, respiratory rate; SBP, systolic blood pressure; SpO_2_, saturation of peripheral oxygen; TG, triglyceride; WHR, waist to hip ratio.

* Independent samples *t*-test.

** ANCOVA adjusted by baseline parameters and sex.

The paired samples *t*-test revealed significant improvements in both groups after 3 months in all variables compared to their baseline (all *P*-values < 0.001). The metformin group showed significant improvements in the third month regarding ACT score (20.65 ± 2.382 vs. 19.69 ± 2.364, *P* = 0.035), waist-to-hip ratio (WHR), systolic BP (SBP), diastolic BP (DBP), respiratory rate (RR), O_2_ saturation (SpO_2_), BS, HgA1c, CRP, TG, HDL, LDL, Chol, FEV_1_, and FVC compared to the placebo group based on independent samples *t*-test results (all *P*-values < 0.05), and the differences between the two groups was not statistically significant in terms of body mass index (BMI) (*P* = 0.254), heart rate (HR) (*P* = 0.074), and FEV_1_/FVC (*P* = 0.063) (Table 2). Although the metabolic and anthropometric parameters showed improvements at 3 months stable state within each group, as revealed by paired *t*-tests (all *P*-values < 0.05), the magnitude of these improvements was more robust in the metformin group in comparison to the placebo group after adjustment for baseline parameters and sex, as revealed by the ANCOVA test (*P*-values < 0.001). However, the ANCOVA test did not show any significant difference between the two groups regarding HR (*P* = 0.348), RR (*P* = 0.083), FEV_1_ (*P* = 0.171), and FEV_1_/FVC (*P* = 0.112) (Table 2).

Since both FEV_1_ and FVC are parameters to evaluate pulmonary function and have a correlation with each other, we also performed a multivariate analysis to compare these variables between groups simultaneously. Our results showed that the intervention group exhibited significantly higher FEV_1_ (*P* = 0.014, effect size = 5.6%) and FVC (*P* = 0.001, effect size = 9.2%) after controlling for the confounding effects of baseline parameters and sex.

During the study period, 14 patients (25.5%) in the placebo group and 10 (18.2%) in the metformin group had severe asthma attacks (*P* = 0.489). During the 3-month follow-up period, 5 patients (9.1%) in the placebo group and 3 (5.5%) in the metformin group had been hospitalized due to asthma attacks (*P* = 0.716), and 12 (21.8%) patients in the placebo group and 8 in the metformin group had ED visits (*P* = 0.459). None of the participants in either the metformin group or the placebo group showed any major adverse medication-related side effects during the follow-up period.

## Discussion

The present study aimed to investigate the effects of metformin on BS control, CRP levels (as an inflammatory marker), ACT score, PFT including FEV_1_, FVC, and FEV_1_ to FVC ratio, and clinical outcomes in patients with concurrent asthma and MetS. Our findings showed that the BMI, WHR, SBP, DBP, SpO_2_, BS, HbA1c, CRP, TG, HDL, LDL, Chol values, FEV_1_, and FVC parameters, and ACT scores had more improvements in the metformin group compared to the placebo group.

Previous studies were mainly conducted on patients with both Asthma and T2DM and assessed the effect of metformin on this population; however, in the present study, we evaluated patients with concurrent asthma and MetS, and to the best of our knowledge, this is the first study that evaluates the effect of metformin on respiratory and metabolic profile of this unique population. Francesca Polverino *et al*^[22]^ demonstrated that metformin leads to airway remodeling, reduced glomerular contraction, and reduced oxidative stress, thereby potentially improving pulmonary and renal indices, including SpO_2_. The pattern of changes in SpO_2_ in our study also indicated that metformin could have a positive effect on SpO_2_, thereby enhancing its levels. Additionally, the changes in FEV_1_ and FVC in our study demonstrated that metformin was able to lead to a reduction in these values. Since metformin also caused statistically significant reductions in BMI and WHR compared to the placebo group, the improvements in FEV_1_ and FVC may be attributed to metformin’s impact on visceral fat^[23]^, and its subsequent effect on decreasing intra-abdominal pressure, improving diaphragmatic function and ventilation efficiency^[24,25]^.

Although the improvement in vital signs and spirometric parameters at the 3-month stable state follow-up visit in both groups can somehow be expected, the degree of these improvements was more robust in the metformin group versus the placebo group. Concerning the reversible nature of PFT parameters in asthmatic patients and the effects of asthma attacks on PFT^[26]^, the evaluation of these parameters at severe asthma attacks for the baseline comparison may be one of the limitations of the present study and further research to elucidate and compare PFT parameters in stable-state disease may further clarify the effects of metformin on PFT of Asthma and MetS patients.

In a cohort study by Wu *et al*^[27]^ on patients with glycemic dysfunction, metformin use was associated with reductions in the hazard of both asthma-related ED visits and hospitalizations. Similarly, in other cohorts of patients with asthma and diabetes, the results showed that metformin led to a reduced risk of attacks, ED visits, and hospitalizations^[15,16]^. Nevertheless, a meta-analysis of observational studies in the diabetic population showed that metformin was associated with a reduced risk of asthma attacks, ED visits, and hospitalizations in patients with concurrent asthma and diabetes^[28]^. Our findings similarly showed a trend toward improvement in asthma-related ED visits or hospitalizations; however, the difference was not of statistical significance, possibly due to the current study’s smaller sample size and shorter follow-up period compared to the mentioned cohort. However, our findings showed that the ACT score was better in the metformin group at the end of the follow-up period.

Regarding inflammatory markers, our findings showed that metformin caused significant reductions in CRP levels compared to placebo, showing evidence of its anti-inflammatory effect. CRP may not be a precise measure of inflammation; however, in the present study, we excluded patients taking oral corticosteroid medications or those with major conditions that alter CRP levels to minimize their impact. Additionally, CRP is more accessible in clinical practice. Nevertheless, in future studies, more accurate inflammatory cytokines could be used to assess the inflammation state in this population.

**Figure 2.**
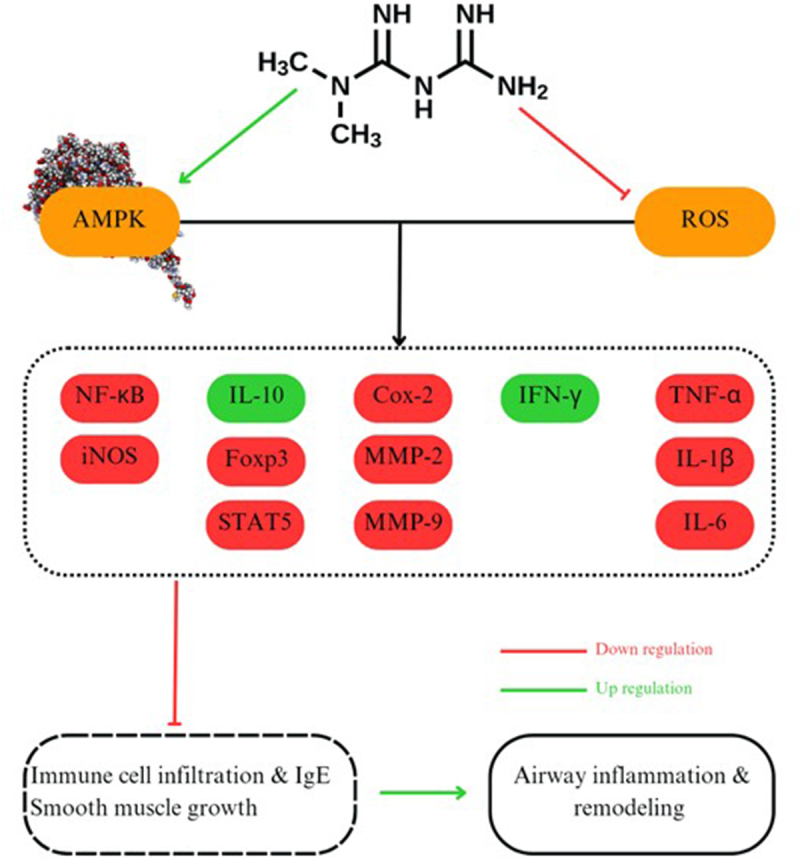
A summary of molecular modifications by metformin in order to reduce airway inflammation and remodeling.

High baseline CRP levels in the metformin and placebo groups can be attributed to MetS and asthma attacks ^[29–31]^. Some studies investigating the anti-inflammatory effects of metformin have shown that this specific medication has the ability to reduce allergic eosinophilic airway inflammation, remodeling, and airway smooth muscle cell proliferation through activation of AMPK, inhibition of reactive oxygen species production, and modification of inflammatory cytokines and nuclear factor kappa-light-chain-enhancer of activated B cells in lung tissue^[32–35]^. Tumor necrosis factor alpha (TNF-α) is a Foxp3 downregulator that leads to suppressive effects on regulatory T-cell (Treg) function^[36]^. Tregs are immune system modifiers that promote interleukin-10 (IL-10) production^[37,38]^. IL-10 is an effective inhibitor of several proinflammatory cytokines. Metformin reduces TNF-α, leading to upregulation of Foxp3, Tregs, and IL-10, which causes reduced airway inflammation^[39]^. Metformin also increases interferon-gamma (IFN-γ), which inhibits the accumulation of eosinophils through suppression of CD4 + T-cell infiltration and decreases IL-4 and IL-5 and IgE production, thus leading to attenuation of inflammation (Fig. 2) ^[39–41]^.

The effects of metformin have also been studied on other obstructive lung diseases, especially COPD. Recent studies suggest that in patients with concurrent T2DM and COPD, metformin could reduce the risk of adverse clinical outcomes such as acute attack, COPD-related hospitalization, and mortality^[42–44]^. The mechanisms behind these effects are not well understood yet; however, studies suggest that metformin could provide anti-inflammatory effects via inhibition of amiloride-sensitive epithelial sodium ion channel (ENaC), reduction in IL-6 and IL-8 levels, and activation of AMPK, nuclear factor erythroid-2-related factor-2 (Nrf2) phosphorylation, heme oxygenase-1, and Wnt/β-catenin pathway in human bronchial cells^[45–47]^. However, in clinical practice, results were contradictory as many studies also reported negative or insignificant effects from metformin on COPD. For instance, a nationwide observational cohort study illustrated that among individuals diagnosed with T2DM and COPD, the utilization of metformin was linked to increased probabilities of bacterial pneumonia, COPD-related hospitalization, and requiring invasive mechanical ventilation^[48]^. In contrast, a retrospective cohort study of 4231 patients with COPD showed that metformin administration in this population may lead to reduced risk of mortality^[49]^. Altogether, the effects of metformin on respiratory outcomes and metabolic parameters of obstructive airway diseases have yet to be further clarified, and future studies could also aim to investigate the effects of this medication on patients with concurrent COPD and MetS.

### Limitations and future directions

The study used CRP as the primary inflammatory marker, which is a nonspecific indicator of inflammation. More precise markers of airway inflammation (e.g., IL-6, TNF-α, and eosinophilic counts) could provide deeper insights into the anti-inflammatory effects of metformin. Incorporation of proteomic or transcriptomic analyses could help identify novel inflammatory pathways influenced by metformin.

The study population was recruited from a single center in Iran, which may limit the generalizability of the findings to other ethnic or geographic populations. The diverse regional demographics in Mazandaran, while a strength within Iranian populations, also introduce variability that may not be representative of broader international populations. Future studies should be conducted across different regions (e.g., North America, Europe, and East Asia) to assess whether metformin’s effects vary by genetic, environmental, or lifestyle factors.

While the observed enhancements in vital signs and PFTs at the 3-month stable-state follow-up were anticipated in both groups, the metformin group demonstrated significantly more pronounced improvements compared to the placebo group. However, given the known variability of PFTs in asthma, particularly their reversible nature and susceptibility to acute exacerbations, our baseline assessment during severe asthma attacks could represent a study limitation. Future investigations should evaluate PFTs during stable disease conditions to better delineate metformin’s effects on pulmonary function in patients with concurrent asthma and MetS.

Also, the study could not control for dietary habits or physical activity, which could independently affect metabolic and respiratory outcomes. Future studies should implement food frequency questionnaires or dietary logs to control for confounding effects of nutrition and use wearable devices (e.g., accelerometers) or validated activity surveys to account for exercise’s impact.

### Novel contributions to existing literature

Prior studies focused on metformin’s effects in asthma patients with diabetes. This is the first DBRCT to evaluate metformin in non-diabetic patients with concurrent asthma and MetS, isolating MetS-specific effects. Our study also simultaneously measures lung function (FEV_1_, FVC, and ACT), metabolic parameters (lipids, HbA1c, and CRP), and clinical end-events (exacerbations and ED visits). Prior work focused on either metabolic or respiratory outcomes.

### Take home message

Metformin benefits non-diabetic MetS-asthma patients by improving asthma control (↑ACT scores), lung function (↑FEV_1_/FVC), oxygen saturation, and metabolic health (↓CRP, lipids, and BP). Clinicians could consider metformin for asthma patients with MetS, even without diabetes. However, further clinical trials are needed.

## Conclusion

Our results showed that administering metformin to patients with concurrent asthma and MetS improves patients’ vital signs, body composition parameters, short-term and long-term BS control, and lipid profile indices. Moreover, it results in a notable decrease in CRP levels as an inflammatory marker and causes improvements in ACT score, FEV_1_, and FVC, contributing to enhanced pulmonary function. Although the metformin group showed a trend toward reduction of severe asthma attacks, ED visits, or hospitalization rates, the difference was not statistically significant compared to the placebo group in the current study. Larger studies with long-term follow-up and further characterization of T2 versus non-T2 inflammation may reveal endotypes of the patients who might probably benefit more.

### Declaration of AI use

During the preparation of this work, the authors used “Grammarly” in order to improve the language proficiency of the manuscript on 7 June 2025^[50]^. The authors confirm that all inputs were de-identified and compliant with GDPR/HIPAA. After using this tool/service, the supervising authors (Dr. Hossein Mehravaran and Dr. Erfan Ghadirzadeh) reviewed and edited the content as needed. We confirm that all authors reviewed the final content and take full responsibility for the content of the publication. Additionally, the authors disclose no conflicts of interest or financial ties to AI vendors.

## Data Availability

The data are available upon reasonable request from the corresponding author. The trial protocol can be accessed at https://en.irct.ir/trial/62400.

## Research registration unique identifying number (UIN)

This study was registered with the Iranian Registry of Clinical Trials (IRCT20220305054199N1, 1 November 2022).
